# Robust consistent single quantum dot strong coupling in plasmonic nanocavities

**DOI:** 10.1038/s41467-024-51170-7

**Published:** 2024-08-09

**Authors:** Shu Hu, Junyang Huang, Rakesh Arul, Ana Sánchez-Iglesias, Yuling Xiong, Luis M. Liz-Marzán, Jeremy J. Baumberg

**Affiliations:** 1 Nanophotonics Centre, Cavendish Laboratory, University of Cambridge, Cambridge, UK; 2 Department of Physics, College of Physical Science and Technology, Xiamen University, Xiamen, China; 3 CIC biomaGUNE, Basque Research and Technology Alliance (BRTA), Basque Research and Technology Alliance (BRTA), Donostia-San Sebastián, Spain; 4 Ikerbasque, Basque Foundation for Science, Bilbao, Spain

**Keywords:** Quantum dots, Nanophotonics and plasmonics

## Abstract

Strong coupling between a single quantum emitter and an optical cavity (at rate Ω) accesses fundamental quantum optics and provides an essential building block for photonic quantum technologies. However, the minimum mode volume of conventional dielectric cavities restricts their operation to cryogenic temperature for strong coupling. Here we harness surface self-assembly to make deterministic strong coupling at room temperature using CdSe/CdS quantum dots (QDs) in nanoparticle-on-mirror (NPoM) plasmonic nanocavities. We achieve a fabrication yield of ~70% for single QD strong coupling by optimizing their size and nano-assembly. A clear and reliable Rabi splitting is observed both in the scattering of each nanocavity and their photoluminescence, which are however not equal. Integrating these quantum elements with electrical pumping allows demonstration of strong coupling in their electroluminescence. This advance provides a straightforward way to achieve practical quantum devices at room temperature, and opens up exploration of their nonlinear, electrical, and quantum correlation properties.

## Abstract (web summary)

A bright pure quantum light source is essential for photonic circuit quantum technologies. Here, authors develop a route to consistently couple single quantum dots into plasmonic nanocavities at high yield, achieving strong coupling at room temperature and electrically pumped devices.

## Introduction

Developing photonic quantum technology relies heavily on the on-chip integration of single photon sources^1–3^. Solid state emitters such as nitrogen-vacancy (NV) centers in diamond and quantum dots (QDs) are excellent two-level systems to utilize for single photon sources^4^. NV centers exhibit outstanding photostability but feature low collection efficiency (limited brightness) and are challenging for practical integration into electrically-pumped devices^5,6^. QDs on the other hand offer significant flexibility for on-chip integration and show extremely high quantum yields^7–11^, however they remain limited by poor spectral purity, weak nonlinearity, and low emission rate [~(10 s)^−1^]. An effective solution is to integrate single quantum emitters into an optical cavity with enhanced local density of photonic states, where increased emission rates are obtained due to the Purcell factor^12–16^. More importantly, the system also exhibits strong nonlinearities and photon blockade when the coupling strength Ω/2 exceeds the total loss and can reach the strong coupling regime^12,17–21^, yielding high spectral purity and single photon nonlinear operation.

Typical dielectric cavities support a high-quality factor *Q* thus suppressing losses, but provide a relatively large minimum mode volume *V* ~ (2*πc*/*ω*_0_)^3^ at frequency *ω*_0_ which limits the coupling since Ω ∝ √(1/*V*), thus reaching single-emitter strong coupling only in cryogenic environments^19,22–25^. Plasmonic nanocavities that give mode confinement down to nanometer (and even atomic) scales are a promising platform to implement robust room-temperature exciton-plasmon strong coupling systems with ultracompact size^12,26–29^. A key challenge is to precisely position and orient a quantum emitter in such nanocavities so that the cavity field and dipole couple efficiently. Various strategies have been developed to integrate single quantum emitters into plasmonic nanostructures^16,30–37^, however, the yield and consistency of strong coupling are far from the realm needed to build realistic devices.

Mostly, strong coupling of plasmonic systems is evidenced by two peaks in their scattering spectra, and typically is only shown for a few structures. Unfortunately such peaks can also arise from multimode plasmonic nanocavities, collective optical interactions^38^, inhomogeneous dielectric cavity environments^39^, irregular nanostructure morphologies, and Fano interference^31^ (see Supplementary Notes 1–3), rather than a true splitting of polariton quantum states. It is thus paramount to report statistics on a large number of coupled structures (>100) which is not yet consistently achieved. Energy splitting in photoluminescence (PL) provides less ambiguous evidence of strong coupling but has only been observed in very few studies^31,40,41^. Even these results are ambiguous - for instance tip-enhanced Raman spectroscopy showed two^41^ or four^40^ PL peaks even using the same type of QD (while being impractical for on-chip integration). Surface trap states or dark excitons may also give multiple peaks that are difficult to exclude based only on PL measurements^13,32^. Other studies on plasmonic nanostructures show multiple peaks in both PL and scattering spectra^31,32^, but point out that dark excitons and intermediate states can contribute such features. There thus remains a lack of reliable and consistent single quantum dot-plasmonic cavity strong coupling systems which suitably match to theory.

**Fig. 1:**
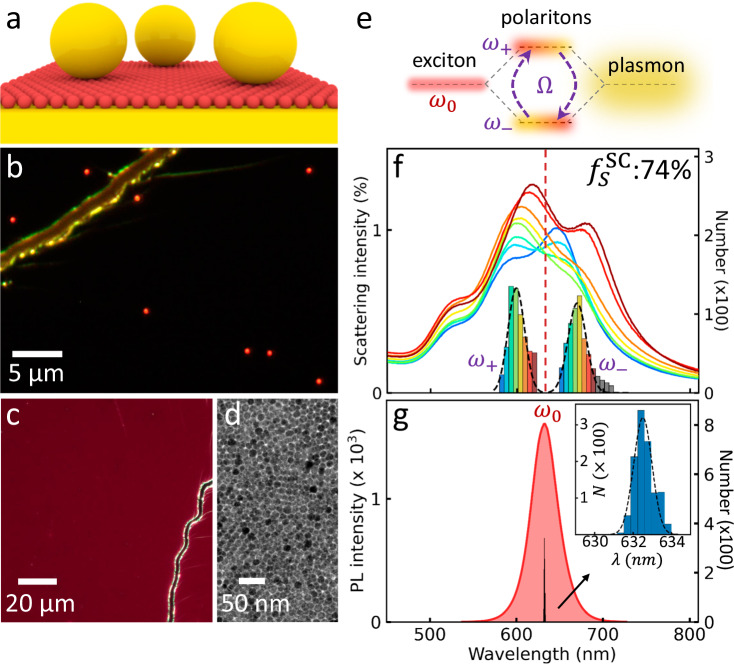
Strong coupling between plasmonic nanocavity and quantum dots (QDs). **a** Schematic and (**b**), dark-field image of monolayer QDs integrated into nanoparticle-on-mirror (NPoM) nanocavities. **c** Dark-field and **d**, TEM images of QD monolayer on (**c**) Si and (**d**) TEM grid. Red color in (**c**) is photoluminescence of QDs under illumination. **e** Exciton (QD) and plasmon (cavity) hybridization forms polaritons in strong coupling regime. **f** Average scattering spectra of >800 NPoMs sorted into bins which all show clear energy splitting of coupled modes. Histogram (black dashed) and average spectra are sorted according to energy of lower (*ω*_−_) and upper (*ω*_+_) polaritons. **g** Typical photoluminescence spectrum (red) of monolayer QD film in (**c**), with spectral distribution of exciton peak at different locations (expanded in inset).

Here, we develop a robust, consistent and simple way to integrate single QDs into a dependable nanocavity termed a nanoparticle-on-mirror (NPoM). Light is tightly trapped inside the nanoscale gap between the underlying Au mirror and the Au NP on top of the QDs^42^. Statistical characterization of thousands of individual nanocavity structures is performed using a sophisticated automated particle identification system. We demonstrate a high fabrication yield of up to 74% room-temperature strong-coupled devices with 200 meV average Rabi splitting, by integrating a close-packed QD monolayer into all NPoMs (Fig. 1). The diameter of each QD is designed to match the facet size of the nanoparticle to enable coupling to a single emitter, and evidenced by the typical blinking behavior. Reliable energy splitting in both PL and scattering are observed, correlated, and well predicted by semi-classical theory. We find that externally exciting either the exciton (by non-resonant optical excitation) or plasmon (by resonant illumination) gives destructive or constructive interference respectively, which induces a different energy splitting in the PL and scattering spectra. Using the plasmonic facets for electrical injection is found to produce single quantum emitter electroluminescence on the verge of the ultra-strong coupling regime (where Ω/*ω*_0_ > 0.2)^43,44^ from both the upper and lower polariton branches, never previously achieved^45,46^. These studies provide a comprehensive understanding of room temperature strong coupling in the single emitter limit, and showcase an ideal system for developing ultra-compact, nonlinear and high-purity quantum light sources that can be integrated into photonic circuits.

## Results and discussion

### Single quantum dot integration in plasmonic nanocavities

Monodisperse CdSe/CdS core/shell QDs of diameter ~12 nm emitting at ~630 nm are assembled into a close-packed monolayer using liquid interface ordering, and integrated into NPoMs (Fig. 1a and Supplementary Fig. 1)^47^. These monolayer QD films exhibit high uniformity under dark field microscopy (Fig. 1b,c), further confirmed by transmission electron microscopy (TEM, Fig. 1d) and PL measurements (Fig. 1g), with emission that is stable in time at ambient conditions. The 80 nm Au nanoparticles (NPs) employed have facet sizes ~20 nm^48^ which can be utilized to fit only a single QD inside each 7 nm-wide cavity mode (Supplementary Fig. 2 and Methods)^49^. Broad area luminescence of the QD monolayer on glass shows highly uniform red emission (Fig. 1c) while the identical red scattering of different NPs on top of the QD film (Fig. 1b) indicate the highly consistent NPoMs with resonance energy matching the QD PL. Statistical characterization of 840 NPoMs shows a clear splitting of the coupled modes (Fig. 1f) at the exciton energy of *ω*_0_ (Fig. 1g). This is due to mixing between plasmon (NPoM) and exciton (QD) states in the strong coupling regime that forms upper (*ω*_+_) and lower (*ω*_−_) polariton states (Fig. 1e) separated by Rabi splitting energy Ω. The yield of distinctively split NPoMs in scattering is as high as ${f}_{S}^{SC}$= 74%, implying near hundred-fold improvement in integration consistency compared to sub-%-level state-of-the-art^30,31,33^. Even averaged spectra of each histogram bin show clear anti-crossing (Fig. 1f), which is preserved over plasmon detunings that arise from slight variations in Au NP size.

### Rabi splitting of photoluminescence

**Fig. 2:**
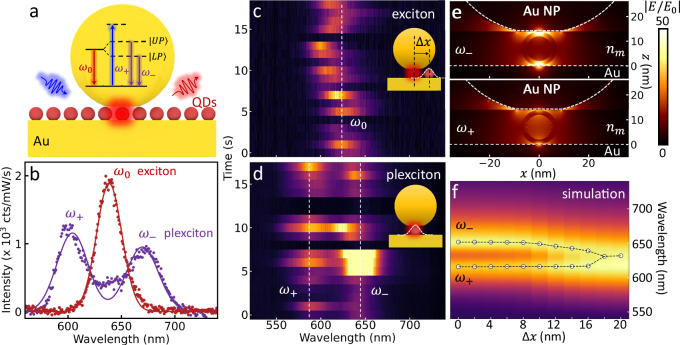
Photoluminescence characterization of single quantum dot (QD) coupled to plasmonic nanocavity. **a** Spontaneous emission process in weak- (red) and strong- (purple) coupling regimes of nanoparticle-on-mirror (NPoM) nanocavity coupled to single quantum dot, under non-resonant pumping (blue). **b** Photoluminescence spectra of weak- (red) and strong- (purple) coupled excitons, selected from measurements on >800 NPoMs. Slow time evolution of photoluminescence spectra of (**c**) exciton- and (**d**) polariton-type NPoMs. **e** Field distribution of single QD-NPoM construct at upper and lower polariton resonances. **f** Simulated scattering spectra of single QD-NPoM as QD is laterally shifted by Δ*x* from center of NPoM (inset in **c**).

To explore strong-coupling luminescence, emission from QDs outside the nanocavities is eliminated by a plasma etching step (see Methods). Employing a 447 nm pump laser to excite PL, a fraction ${f}_{PL}^{SC}$= 33% of NPoMs show a PL energy splitting, while the rest emit at a similar wavelength to the exciton (Fig. 2a, b). This convincingly demonstrates our system reaches the strong coupling regime and excludes confusions from multimode scattering spectroscopy. We note that this ${f}_{PL}^{SC}$= 33% fraction (or >50% of the NPoMs showing DF splitting) is the highest yield so far obtained for emission energy splitting of single QD-plasmonic cavity systems at room temperature, and is already sufficient for practical optoelectronic devices. Compared to alternative systems such as NV centers^5^, these emitters approach identical performance. The near equal emission intensity from the upper and lower polaritons states shows that thermalization is not achieved in these systems ($\Omega \gg {k}_{B}T$), suggesting that relaxation from high energy pumped states equally populates both states, and that Purcell-enhanced radiative recombination is much faster than any other relaxation. One complication of the resulting sub-ps emission time is that g^(2)^ measurements become inaccessible.

Blinking and wavelength fluctuation of PL from these NPoMs are seen in both weak (Fig. 2c) and strong (Fig. 2d) coupling regimes, which is a characteristic that confirms the emission from single QDs. This is less pronounced than for single QDs on glass, likely because the surrounding Au removes and screens trapped charges. The constant Ω during blinking confirms coupling of single QDs to the nanocavity is preserved, but the upper polariton emission intensity fluctuates less than the lower polariton. To understand this, the field distribution of single QD-NPoM constructs is simulated at both upper and lower polariton resonances (Fig. 2e, Methods). At *ω*_−_, the intense field is strongly confined in the QD, in contrast to the much less confined field at *ω*_+_ (Supplementary Fig. 3). The lower polariton is thus more sensitive to fluctuations in charge trapping at the QD surface^13^.

Compared to dyes or transition-metal dichalcogenides (TMDs) that have oriented dipoles, the spherical QD symmetry ensures the dipoles co-align with the optical field which is near-perpendicular to the metal facets. As a result, instead of orientation effects, we suggest that weakly-coupled NPoMs mainly arise from reduced mode overlap when no QD is exactly located inside the 7nm-side near-field mode under the NPoM facet (Fig. 1c,d). Simulating the scattering spectra when continuously shifting the QD away from the NPoM center (Fig. 2f) shows that instead of a gradual reduction in Ω (tracking the mode overlap), the strong coupling barely changes for Δ*x* < 8 nm and collapses only for Δ*x* > 16 nm. The spatial maps (Supplementary Fig. 4) show how the optical fields are pulled laterally by the QD due to its high reflective index, and thus maintain high coupling to the cavity mode despite the lack of deterministic positioning of each QD. While estimated yields combining this with the observed QD separations are ~80% (Supplementary Note 2), contributions also arise from variations in NP shape and facet size.

### Anti-crossing of scattering and photoluminescence

**Fig. 3:**
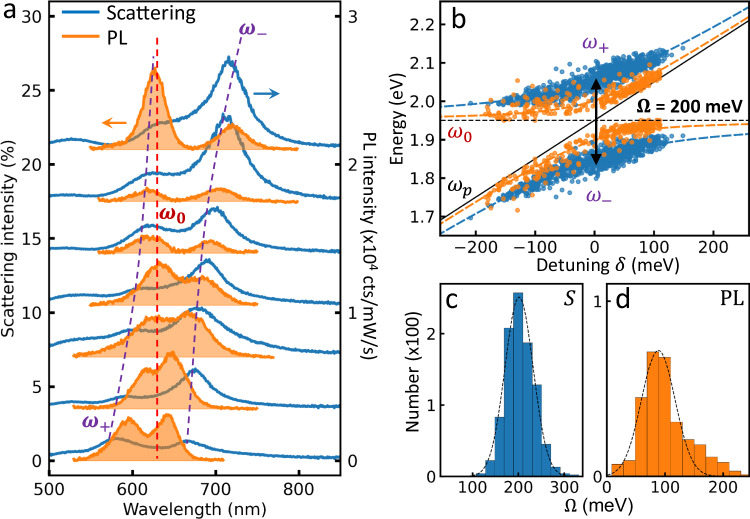
Anti-crossing of photoluminescence and scattering in the strong coupling regime. **a** Correlation of typical photoluminescence (orange) and scattering (blue) spectra of 7 nanoparticle-on-mirror (NPoM) nanocavities *vs* cavity detuning. **b** Extracted anti-crossing curve of polaritons (see text). Rabi splitting energies extracted from (**c**), scattering spectra ( > 600 NPoMs), and (**d**), photoluminescence (>350 NPoMs).

The high yield allows us to extract a reliable correlation for the detuning dependence $\delta={\omega }_{p}-{\omega }_{0}$ (Fig. 3a) thus providing deeper insight into the fundamentals of strong coupling in the single QD limit. Since the QD emission peak varies by <0.2% (Fig. 1g inset), each measured (*ω*_+_,*ω*_−_) can be inverted from $2{\omega }_{\pm }=({\omega }_{p}+{\omega }_{0})\pm \sqrt{{\Omega }^{2}+{\delta }^{2}}$ to give (*ω*_p_, Ω) using the simple two coupled-oscillator model^12^. Across 900 NPoMs, both scattering and PL polariton peaks show a clear anti-crossing *vs* cavity detuning (Fig. 3b), with Ω_PL _< Ω_*S*_. The average Rabi splitting Ω_*S*_ ~200 ± 45 meV obtained (Fig. 3c) satisfies the criteria for strong coupling, $\Omega > \bar{\gamma }=(\kappa+\gamma )/2$ ~ 120 meV (Supplementary Fig. 5) for cavity linewidth *κ* and exciton linewidth *γ* (note alternative definitions using Ω/2), as well as approaching ultra-strong coupling since maximum Ω/*ω*_0_ ~ 0.2^21^. On the other hand, the exciton splitting Ω_*PL*_ is half this (Fig. 3b,d), emphasizing how defining Rabi splittings based on characterizing only scattering or photoluminescence is misleading. Although two peak features in photoluminescence and scattering have been provided in prior studies to evidence strong coupling^30–33,40,41^, this conclusion relies on selecting spectra which can also be obtained here from aberrant structures (see detailed discussion in Supplementary Note 3). Clear and statistically robust correlations in energy splitting between nanocavity (scattering) and emitter (PL) have not yet been reported.

### Semi-classical simulation

**Fig. 4:**
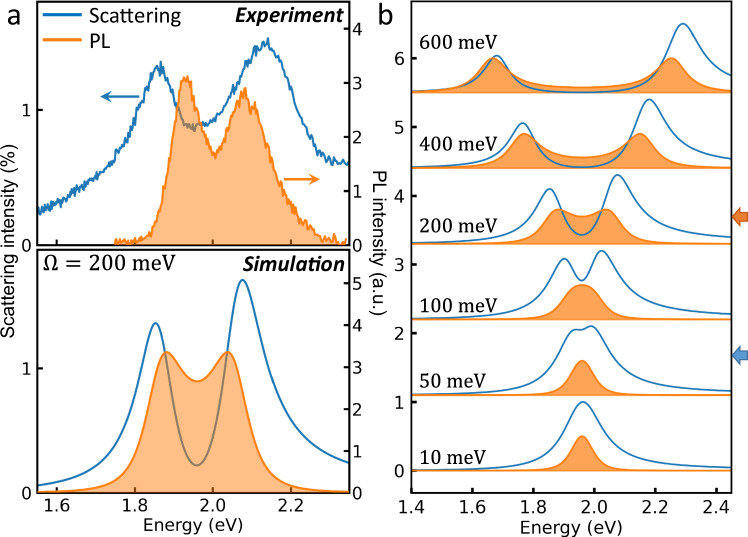
Comparison between experiment and simulation of single quantum dot strong coupling. **a** Experimental (top) and simulated (bottom) scattering and photoluminescence spectra of single quantum dot-plasmonic nanocavity in strong coupling regime at near zero detuning. **b** Simulated scattering and photoluminescence spectra of single quantum dot in plasmonic nanocavity for increasing coupling strengths shown.

To track this interplay of cavity and exciton loss in the strong coupling regime, we simulate scattering and PL spectra of single QD-plasmonic cavity strong-coupling based on a semi-classical treatment^31^ using parameters extracted from experiment (Supplementary Fig. 5). The two oscillators are coupled with strength Ω = 200 meV, giving a good match between experiment and theory (Fig. 4a). Keeping the losses fixed, gradually increasing Ω shows different onsets of energy splitting in PL and scattering (Fig. 4b, arrows). The scattering spectra split before the coupling strength meets the criterion of strong coupling, because of destructive Fano interference between exciton and plasmon^31^. This interference is instead constructive in PL, as pumping excites the exciton component of both polaritons. This difference between Ω_*PL*_ and Ω_*S*_ disappears at higher coupling strengths $\Omega > 2\bar{\gamma }$, because the polariton spectral overlap becomes minimal (Supplementary Fig. 10). Related results have been obtained previously in the context of quantum well-microcavities^50^, where the energy splitting in PL depends on Rabi splitting and damping contributions, but only matches for well-separated peaks. Energy splittings in PL spectra are thus more stringent to distinguish strong coupling than scattering, particularly for smaller coupling strengths. Corresponding effects are seen as the cavity damping rate (*κ*) is varied (Supplementary Fig. 11). This result also explains why the yield of splitting in PL is less than in scattering since the PL splitting is difficult to distinguish in our regime (Fig. 3c,d) where Ω is relatively small.

### Electrically pumped device in strong coupling regime

**Fig. 5:**
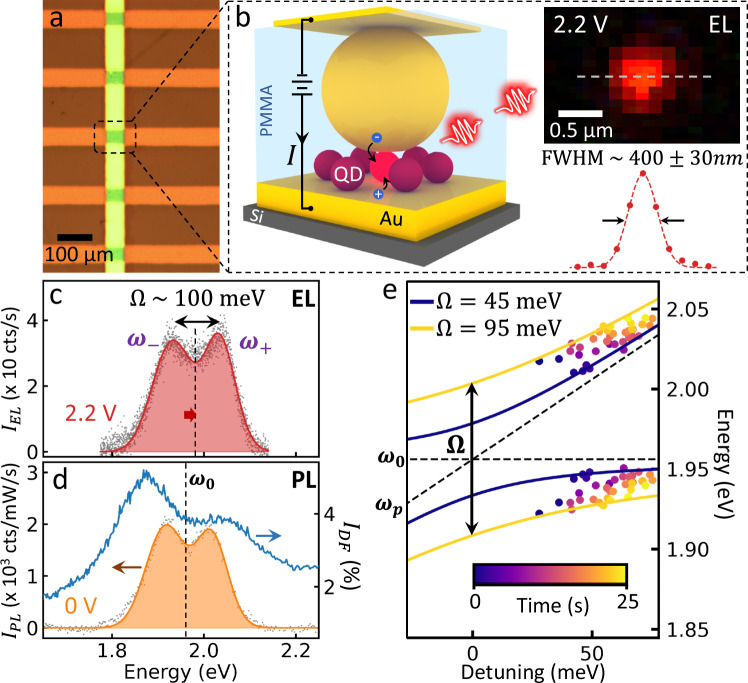
Electrically-pumped strong coupling. **a** Device crossbar geometry. **b** Crossbar contact geometry eliciting light emission from electrically-pumped NPoM nanocavity at 2.2 V. The full width half maximum (FWHM) of the electroluminescence image (inset) is 400 ± 30 nm, as predicted from the diffraction limit *λ*/2*NA* ~ 390 nm. Emission spectra under (**c**) optical and (**d**) electrical excitation. **e** Evolution of polariton modes over time as the plasmon mode blue-shifts during facet reshaping.

To confirm the capability for integration, we show this NPoM geometry is amenable to electrical pumping by tunneling injection. After fabrication of NPoMs on top of 50 µm-wide Au stripes, an insulating PMMA layer is spun over the devices and etched back to expose the top of the NPs so that semi-transparent 12 nm-thick Au crossbar lines can be evaporated on top (Fig. 5a, b, details in Methods and Supplementary Fig. 12). Each junction forms a back-to-back Schottky diode over the applied voltage range from −1.5 to 1.5 V (Supplementary Fig. 13a), where the observed current of a few nA/V is expected for a single NPoM device. The resulting devices switch on at ~2 V bias, as expected for the first exciton energy of these CdSe QDs, and light emission is observed from a diffraction limited spot at the randomly located dominant NPoM within each crossbar overlap (Fig. 5b)^51^. This electrical contacting geometry was first used for molecular emitters^51^, but restricted to the weak coupling regime with ~200 molecules coupled inside the nanocavity. The crucial grand challenge remains to achieve strong coupling and electrically-pumped light emission from such junctions.

Measuring the electroluminescence (EL) spectra indeed shows the same strong-coupling split polariton peaks as in PL spectra. As above, spectral wandering of the EL emission over time confirms that a single QD is emitting in the cavity (Supplementary Fig. 14). At a constant DC bias of 2.2 V, average currents of 0.6 µA are seen (Supplementary Fig. 13b), although this also fluctuates over time. Even in these non-optimized structures where the collection geometry is restricted, 10^5^ counts/s can be obtained corresponding to the emission of ~1 photon/cavity lifetime. While the ability to electrically inject electrons and holes from both Au contacts is viable, it is not yet optimal as no hole- or electron-blocking layers have been included due to their potential impact on the plasmonic emission efficiency (>50%)^52^. Future work will need to include such layers to increase the injection efficiencies. Our results however confirm that single QD-NPoM strong coupling at room temperature can be driven in an electrical device, opening the way to CMOS integration. A key issue to solve is that these devices are not stable for long operation under electrical pumping, as the Au facet atoms can migrate (also seen for NPoM optical pumping^27^) leading to device breakdown. In many cases this leads to facet reshaping and bridging which blue-shifts the plasmon mode (Fig. 5e), moving out of the zero-detuning situation. Due to this, statistics on the Rabi coupling from EL are not yet robust. A variety of strategies such as embedding the QDs in a solid polymer are under investigation to prevent atomic migration.

In conclusion, we established a route to create robust and consistent coupling of single QDs inside individual NPoM nanocavities. We achieved a high device yield of 74% strong coupling constructs, far beyond any previous strategies, and with a large Rabi splitting energy of 200 meV at room temperature. Through systematic studies on more than a thousand individual nanocavities, we track strong coupling in the single quantum emitter limit. We show that upper polariton emission is stable at room temperature and present the first reliable correlation of the energy splittings of PL and scattering spectra in the near ultra-strong coupling regime. We demonstrate that such single quantum emitter strong coupling is accurately predicted using semi-classical theory, and attribute the different splittings to destructive *vs* constructive quantum interference of plasmons and excitons. We demonstrate that this NPoM geometry is amenable to simple electrical pumping, which gives strong-coupled quantum LEDs. Our findings give a comprehensive insight into light-matter strong coupling in the single emitter limit at room temperature and provide a viable route to robust, integrable, and nonlinear single photon sources with high brightness and purity using electrical pumping.

## Methods

### Monolayer quantum dot assembly and integration into NPoMs

CdSe/CdS core/shell quantum dots (QDs; Fraunhofer Ltd) with a diameter of 12 nm and oleic acid ligand coating of 2 nm are diluted 100 times to 0.1 mg/ml with hexane before used for assembly. The QDs are assembled into monolayers using a liquid-air interface assembly technique^47,53^. As the schematic shown in Supplementary Fig. 15, 2 mL diethylene glycol (DEG) is added to a glass petri dish with a 150 µL drop of 0.1 mg/ml QD in hexane solution deposited on top. The container is then immediately covered with a glass lid and the hexane slowly evaporated over 10 min. The lid is taken off for another 10 min to ensure the complete evaporation of hexane. The monolayer QD film forms at the interface and is transferred by gently touching the film to a Au-coated substrate which is mechanically lowered into contact. Finally, the substrate is rinsed with ethanol to remove the extra DEG on the surface and dried with nitrogen gun. The whole processes should be implemented in a quiet and dark space to minimize the disturbance from the environment. The layer of QDs can be tuned from single- to multi-layer by changing the volume of QD solution or repeating the processes.

For NPoMs, 80 nm diameter Au NPs are used (BBI Solution). The NPs are centrifuged twice and cleaned with distilled water to remove additional citrate surfactant. Then 30 µL NP solution is deposited onto the monolayer QD substrate for 30 s, which is then rinsed with water to form NPoMs. The fabricated substrate is treated with O_2_ plasma for an hour to quench the emission of QDs outside the NPoMs (Supplementary Fig. 16).

### Characterization of monolayer quantum dot substrate and NPoMs

The average diameter of the QDs is measured as 12 nm (Supplementary Fig. 1a) using transmission electron microscopy (TEM). The QD films can be transferred on scales of centimeters (Supplementary Fig. 1b, inset), and are continuous and highly uniform under dark field microscopy as well as TEM (Fig. 1d). The height of the QD film is measured to be 14 nm (Supplementary Fig. 1c, d) using atomic force microscopy (AFM), combining the size of each QD (12 nm) and its ligand shell (1 nm).

The photoluminescence (PL) and scattering measurements on single particles are obtained in a home-built automatic particle tracking rig. In the scattering measurements, a white light (halogen lamp) illuminates at high angle through the outer ring of the dark field objective lens (Olympus × 100 NA0.9) while the scattered light is collected at lower angles through the main inner path. The collected scattered light then passes through a multi-mode fiber to reduce the collection area to a confocal spot and is recorded on a spectrometer (Ocean Optics QE Pro). For PL measurements, the sample is pumped through the same lens with a 447 nm laser and the signal is collected over the same collection angles.

Supplementary Fig. 2a shows the computed lateral field distribution of the lowest dominant (10) plasmon mode that couples with the QD, where the field is tightly-localized at the center of the facet with a spatial extent close to 10 nm. Although the typical facet diameter of NPoMs is two QDs across (Supplementary Fig. 2b), efficient coupling only occurs when the QD is positioned near the center of the cavity.

### Electroluminescent LED device fabrication

The LED devices are fabricated according to previous methods^51^. In brief, the bottom Au patterned stripes of 50 nm thickness are formed by Au evaporation through a patterned mask (Supplementary Fig. 12). A monolayer of QDs and then Au NPs are constructed on top using the assembly methods above. An insulating layer of PMMA is spin-coated and then etched back with O_2_ plasma to expose the upper facet of the Au NPs for contacting. Finally, 12 nm-thick Au stripes are evaporated on top through the mask (rotated by 90°) to create the crossbar contacts. In this device geometry, charge transport can only take a path through NPoM junctions defined by the QD monolayer.

### FDTD simulation

The diameter of the Au nanoparticle is 80 nm with a bottom facet of 20 nm (as in expt). The QD contains a CdSe core of 10 nm diameter and a (non-resonant) CdS shell of 2 nm thickness and is embedded in a uniform dielectric medium with reflective index of 1.7. Light illuminates the NPoM in the horizontal direction with TM polarization. The exciton oscillator strength and background dielectric constant of CdSe are shown in Supplementary Note 1, and the Johnson & Christy dielectric function for Au was used. A refined mesh of 0.2 nm was used in the region of the gap and 5 nm into the gold particle and mirror.

### Supplementary information

Supplementary Information

Peer Review File

## Data Availability

The data that support the findings of this study are available from the corresponding author and the data is deposited in the Cambridge Open Data archive at 10.17863/CAM.110642.
