# Supplementary material for: Robust consistent single quantum dot strong coupling in plasmonic nanocavities
Source: Nat Commun. 2024 Aug 9;15:6835. doi: 10.1038/s41467-024-51170-7 (PMC11315915; doi:10.1038/s41467-024-51170-7)
Supplement: Supplementary file 1 — Supplementary Information [file 41467_2024_51170_MOESM1_ESM.pdf]

Supplementary Information

Robust consistent single quantum dot strong coupling in plasmonic nanocavities

Shu Hu[1,2†*], Junyang Huang[1†], Rakesh Arul[1], Ana Sánchez-Iglesias[3], Yuling Xiong[1], Luis M. Liz-Marzán[3,4], Jeremy J. Baumberg[1*]

[1] Nanophotonics Centre, Cavendish Laboratory, University of Cambridge, Cambridge, CB3 0HE, UK

[2] Department of Physics, College of Physical Science and Technology, Xiamen University, Xiamen 361005, China

[3] CIC biomaGUNE, Basque Research and Technology Alliance (BRTA), Paseo de Miramón 194, Donostia-San Sebastián 20014, Spain

[4] Ikerbasque, Basque Foundation for Science, Bilbao 43009, Spain

[†] these authors contributed equally

* emails: shuhu@xmu.edu.cn, jjb12@cam.ac.uk

Contents of Supplementary Information:

Fig. S1: Sample uniformity, and QD height
Fig. S2: Plasmonic mode and spatial scales of NP facet and QDs
Supplementary Note 1: FDTD simulation details of the QD
Fig. S3: Simulated field profile of upper and lower polaritons
Fig. S4: QD position dependent field distribution of single QD-NPoM constructs
Fig. S5: Parameters extracted from PL and scattering
Supplementary Note 2: Estimate of expected scattering yield
Supplementary Note 3: Data of aberrant PL and scattering spectra (Figs. S6-S9)
Fig. S10: Schematic destructive and constructive interference of plexciton
Fig. S11: Simulated scattering and PL spectra for increasing cavity damping rate
Fig. S12: Schematic fabrication route for electroluminscent NPoM LED devices
Fig. S13: Electrical measurements of NPoM LED device
Fig. S14: Time dependent electroluminescence fluctuations from an active NPoM LED device
Fig. S15: The schematic of liquid-air interface assembly process
Fig. S16: QD photoemission from monolayer QD film with and without NPoM cavity

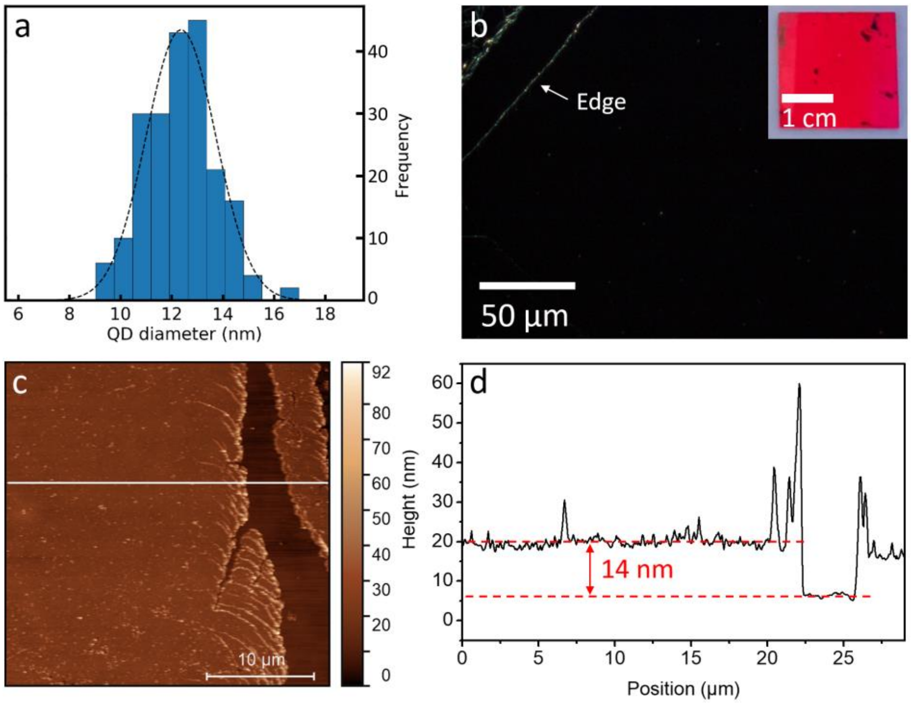

Fig. S1 | The characterization of sample uniformity and QD height. a, Statistics of QD diameter distrbution measured with transmisson electron microscopy (TEM). **b,** Dark field image of monolayer QD film on Au showing high uniformity over large areas. Inset shows the QD film on the centimetre-scale under UV lamp illumination which gives red PL. **c,d,** Atomic force microscopy (AFM) showing (c) mapping and (d) linescan of the monolayer QD film, in the vicinity of a scratch to allow height of the QDs to be extracted.

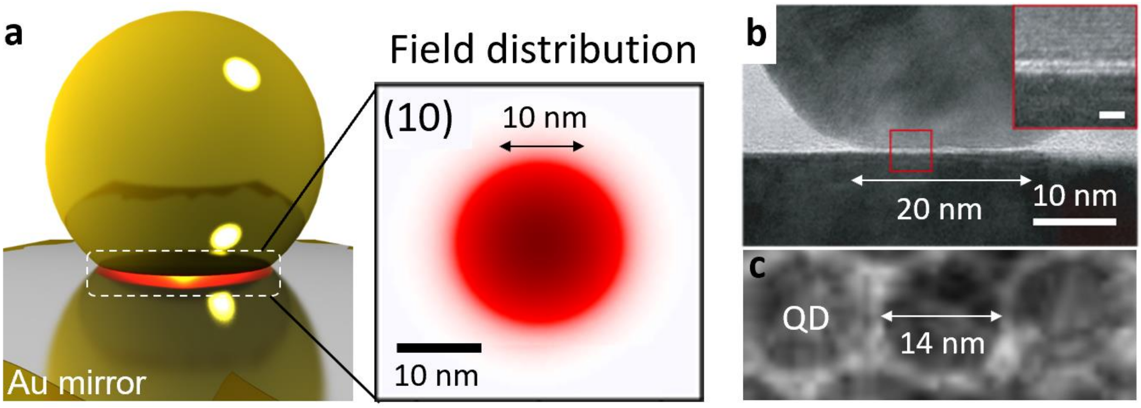

Fig. S2 | Plasmonic mode of of nanoparticle facet and spatial scales of QDs. Size comparison of **a,** optical (10) plasmon mode inside the NPoM nanocavity, and **b,c,** TEMs of typical nanocavity facet (b) and QDs (c) on the same scale. The images in (a) and top TEM image in (b) are adapted from [Q Lin *et al.* Optical suppression of energy barriers in single molecule-metal binding. *Sci. Adv.* **8**, eabp9285 (2022)] and [W Chen *et al.* Probing the limits of plasmonic enhancement using a two-dimensional atomic crystal probe. *Light Sci. Appl.* **7**, 56 (2018)] respectively.

Supplementary Note 1. The dielectric function of the quantum dot in FDTD simulation

The dielectric function (ϵ_{qdot}) of the quantum dot was approximated with a Lorentz oscillator model at the frequency of the exciton. These are comparable to values obtained in the literature[1]:

$$\epsilon_{\text{qdot}} = \epsilon_2 - \frac{A\omega_0^2}{\omega^2 - \omega_0^2 + i\gamma\omega}$$

where ϵ_2 = 2.7^2 is the background dielectric index, A =0.403 is the oscillator strength of quantum dot, γ =90.2 THz is the linewidth of dielectric function, and ω_0 = 2π(471.5) THz is the exciton angular frequency of quantum dot.

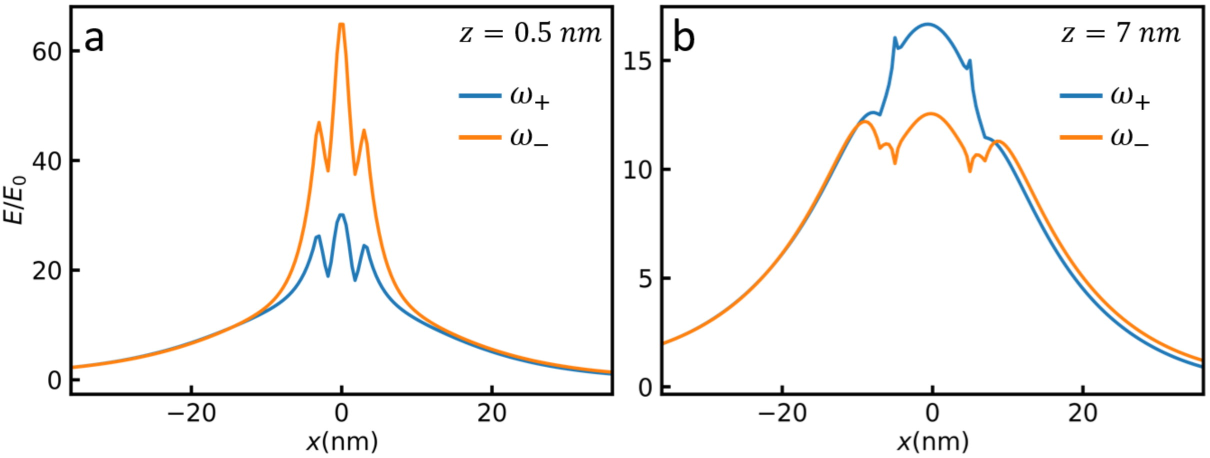

Fig. S3 | Simulated field profile of upper and lower polaritons. Simulated field profile of upper and lower polaritons in the single QD-NPoM construct (Figure 2e) at the center plane and bottom of the gap at heights **a,** just below the QD at 0.5nm and **b,** through the QD centre 7nm.

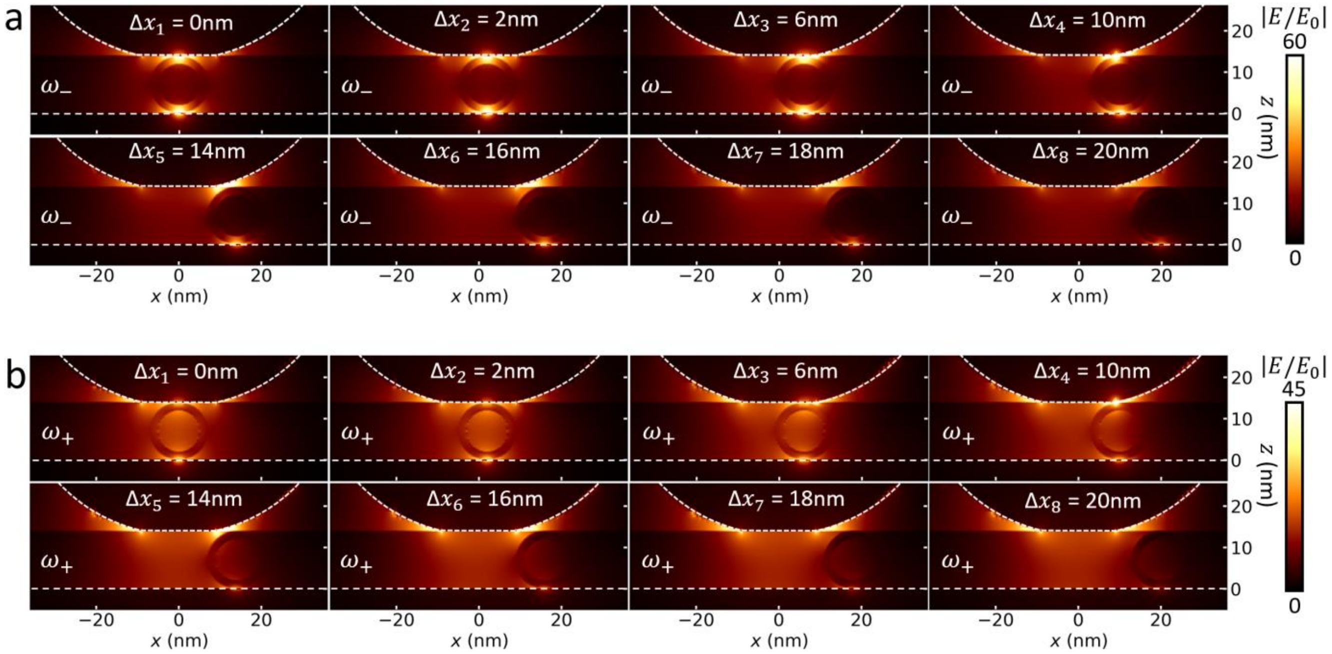

Fig. S4 | QD position dependent field distribution of single QD-NPoM constructs. QD position-dependent field distribution of single QD-NPoM construct as QD shifted sideways (as labelled), at **a** lower and **b** upper polariton resonances.

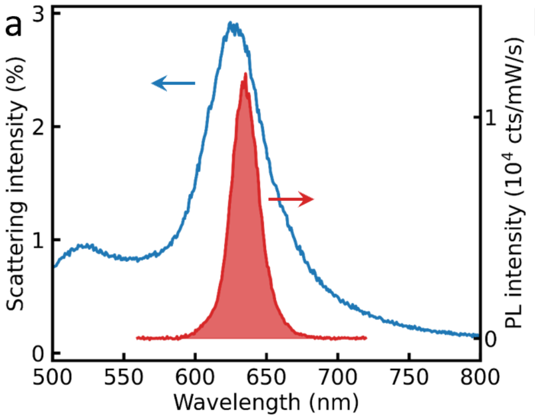

b

Plasmon energy (ω_p)	1.96 eV
Exciton energy (ω_0)	1.96 eV
Damping rate of cavity (κ)	150 meV
Damping rate of QD (γ)	90 meV

Fig. S5 | Parameters extracted from PL and scattering. a, Example of scattering and PL spectra from an NPoM in the weak coupling regime, used for extracting parameters. **b,** Summary of parameters extracted from the scattering and PL spectra.

Supplementary Note 2. Estimate of expected scattering yield

The QDs here are assembled into a close-packed monolayer (Fig.1c,d) which appears to be reasonably uniform across the sample, with average QD centre-to-centre separation of 19$\pm$5nm. Matching the simulation to experimental linewidths suggests that for positions > 9nm from the facet centre, the polariton splitting would be not resolved (as seen from Fig.3c, we take splittings < 100meV as weak coupling). Assuming the NP which is drop cast on top has a random chance of lying in any position, this would imply that the yield of NPoMs in strong coupling would be 79.8% (not far from the 74% measured). However this estimate depends on accurate quantification of the distribution of QD separations, the precise way strong/weak coupling is partitioned, and how the field distribution is further modified with more than one QD in different positions (which compete for field volume), all of which are not easily confirmed. Further modifications are caused by fractions of non-spherical NPs and variations in facet size.

Supplementary Note 3: Data of aberrant PL and scattering spectra

Discussion of prior work: We first note that [34-36] did not achieve strong coupling (despite [36] using two not one QD). [37] discusses coupling to J-aggregates (many molecules and not single QDs) and again shows only a splitting in scattering, rather than in PL. [38] is a useful theory paper that shows that the splitting of scattering spectra is very unreliable to determine the strong coupling. This is reinforced by [39] which did not achieve strong coupling but emphasises that the splitting in scattering spectra cannot be used to demonstrate strong coupling.

Directly relevant experiments: [32] shows two examples of splitting in scattering and PL, reproduced below (Fig. S6). However the authors found they need to use bright and dark excitons (previously unseen) to account for this (dashed lines in Fig. S6c,d), and this confusingly requires coupling to one mode seen in PL, and one mode not seen. They note a 'vast set' of data, and show 5 graphs in the paper & SI which are however less consistent. To compare, we show two cherry-picked spectra from our >800 QD NPoM measurements giving similar data (Fig. S6e,f), which we believe are uncharacteristic because the NP is actually decahedral. We note the electron microscopy in [32] shows all devices have very different nano-morphologies, uncontrolled, and thus poorly suited for basic science or applications, while here QD NPoMs are 74% reproducible.

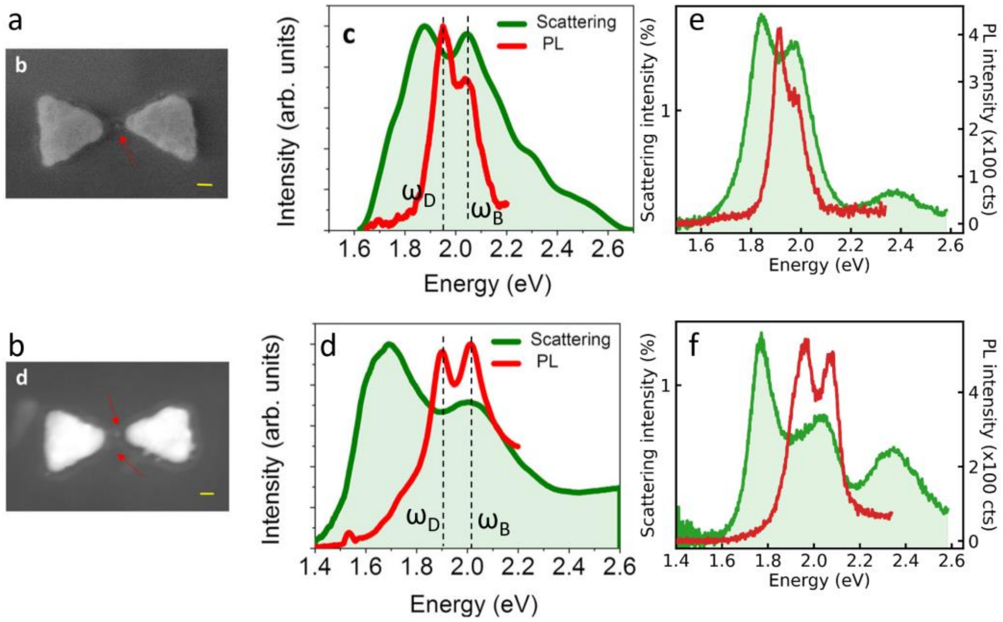

Fig. S6 | Comparison of [32] with selected NPoMs. a,b SEM images of Au bowtie structures with single quantum dot coupled in the metallic gap and their corresponding **c,d** scattering and photoluminescence spectra (from [SN Gupta *et al. Nat. Commun.* **12**, 1310 (2021)]). The splitting of photoluminescence is not correlated with scattering spectra, which was explained by emission from bright (ω_B) and dark (ω_D) excitons (dashed lines). **e,f** Cherry-picked scattering and photoluminescence spectra of two NPoMs with integrated QDs that resemble the data in **c,d**.

[30] and [33] show only selected scattering spectra (five in each case) with two peaks (Fig. S7a,b). Besides the use of scattering rather than PL noted above, we again note potential issues with cherry picking. We thus show various outliers (Fig. S7c) from our full data set where different numbers of peaks, or different spacings are obtained. The key point is that only statistically large data sets can give any meaningful conclusions about these systems. This is a key aspect of the present work.

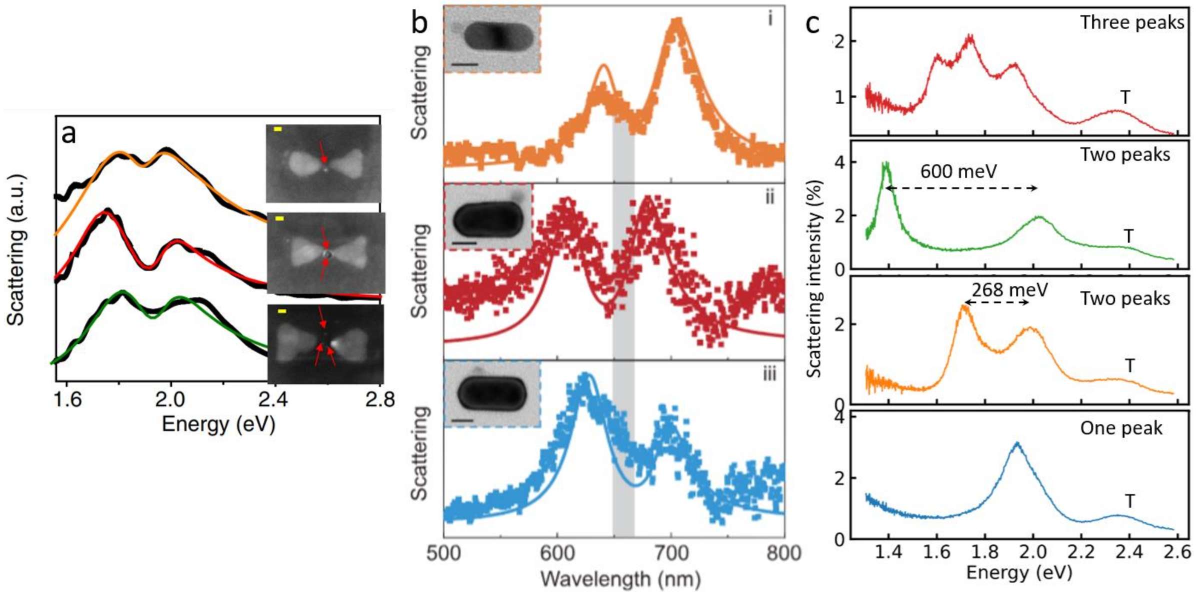

Fig. S7 | Scattering spectra comparison of [30] and [33] with selected NPoMs. a, Scattering spectra of Au bowtie nanostructures with single quantum dot inside the gap (from [K Santhosh *et al*. *Nat. Commun.* **7**, 11823 (2016)]). **b,** Scattering spectra of single nanorods randomly surrounded by quantum dots (from [J-Y Li *et al*. *Nano Lett.* **22**, 4686 (2022)], reprinted in part with permission. Copyright 2022 American Chemical Society). **c,** Cherry-picked scattering spectra of QD NPoMs that show differences in number of peaks and energy separations. T = transverse mode.

[31] shows scattering and PL data which are to evidence strong coupling. However the QDs are randomly attached around the plasmonic nanoparticles (Fig. S8a,b) and not clearly coupled. That they cannot be proven to be responsible for splitting in light emission is shown again by our control data when we omit QDs from the devices (Fig. S8c,d) - these still show exactly the same weak emission that matches exactly the scattering plasmon resonances. The likely explanation here is that this luminescence is from the metal (as seen elsewhere), which is why it is so weak, and slightly enhanced by the plasmonic mode outcoupling.

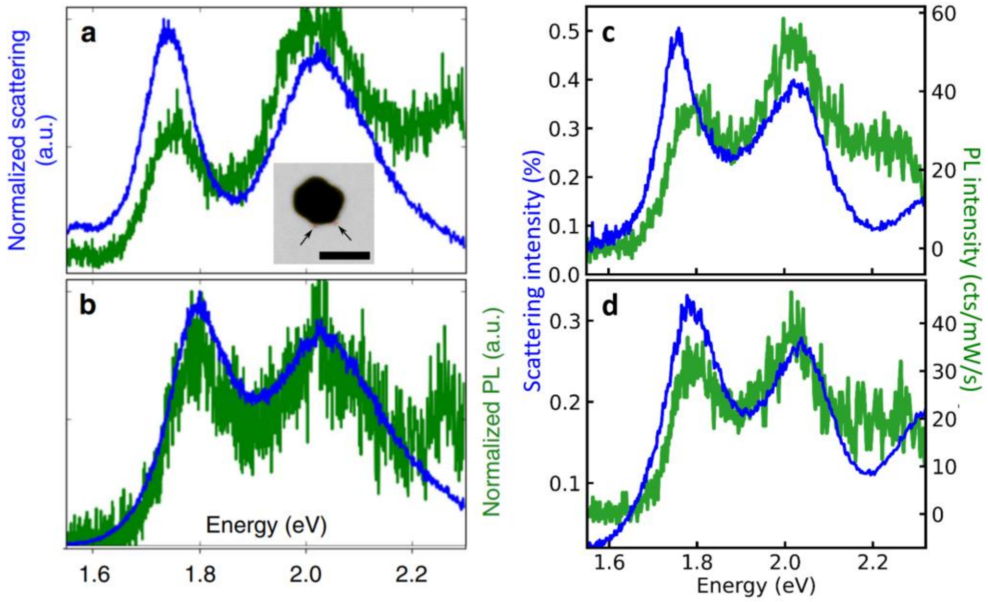

Fig. S8 | Scattering and photoluminescence spectra comparison of [31] with selected NPoMs. a,b Scattering (green) and photoluminescence (blue) spectra of nanoparticles with quantum dots randomly attached (from [H Leng *et al. Nat. Commun.* **9**, 4012 (2018)]). **c**,**d** Cherry-picked scattering (green) and photoluminescence (blue) from NPoMs without QDs (using citrate layer as a spacer).

Finally we examine the opposite regime, where a plasmonic tip is used to probe QDs, as in [41] and [40] while tracking light emission (Fig. S9a,b). In this case no plasmonic mode can be separately measured, making the emission origin harder to confirm. Once again the multiple peak spectra seen can be cherry picked from outliers in our data set (Fig. S9c), which are not the norm.

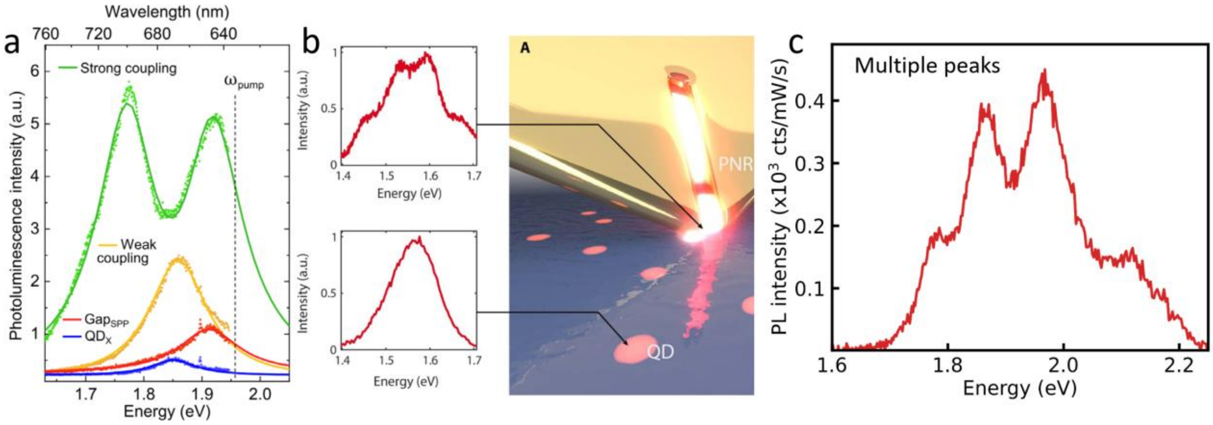

Fig. S9 | Photoluminescence spectra comparison of [40] and [41] with selected NPoMs. a,b Photoluminescence spectra of QDs under a plasmonic tip (from [K-D Park *et al. Sci. Adv.* **5**, eaav5931 (2019), reprinted with permission from AAAS] and [H Groß *et al*. *Sci. Adv.* **4**, eaar4906 (2018), reprinted with permission from AAAS] respectively). **c,** Cherry-picked photoluminescence spectra from a QD NPoM nanocavity showing multiple plasmon mode peaks.

We also note that while such tip based approaches are an experimental tour-de-force (hence rarely reported), they have more slender prospects for further scientific applications. This is why high yield, repeatable and robust results, and electrical driving of NPoM devices are key advances.

In summary, all these prior works are susceptible to selective selection of spectra to identify a few that best match the 'expected' features of strong coupling. What we show here is a route to achieve high yields of precisely defined constructs, allowing us to create electrically pumped devices for obtaining robust polariton emission from single quantum dots for the first time.

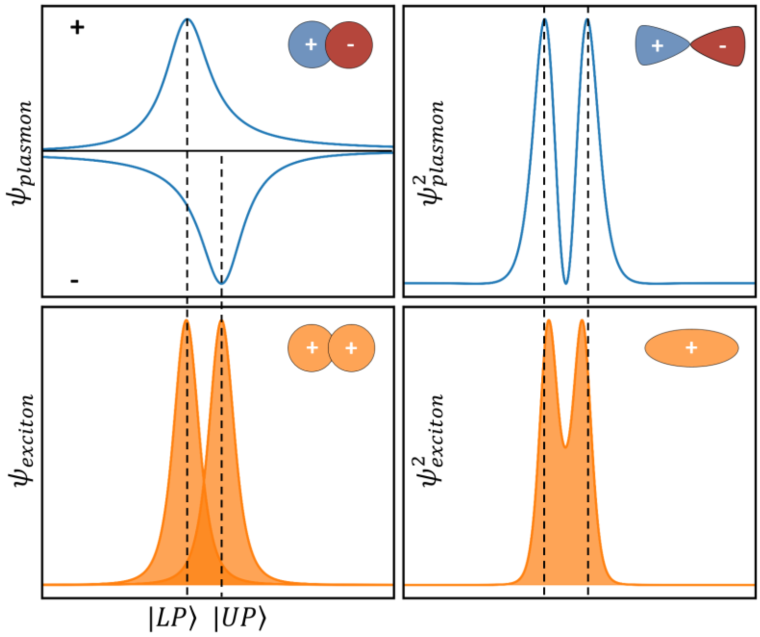

Fig. S10 | Schematic destructive and constructive interference of plexciton. Schematic destructive and constructive interference of plasmon and exciton in the strong coupling regime when probed by PL or scattering (left). The difference in interference phase leads to the difference in extracted energy splitting in scattering and PL spectra (right).

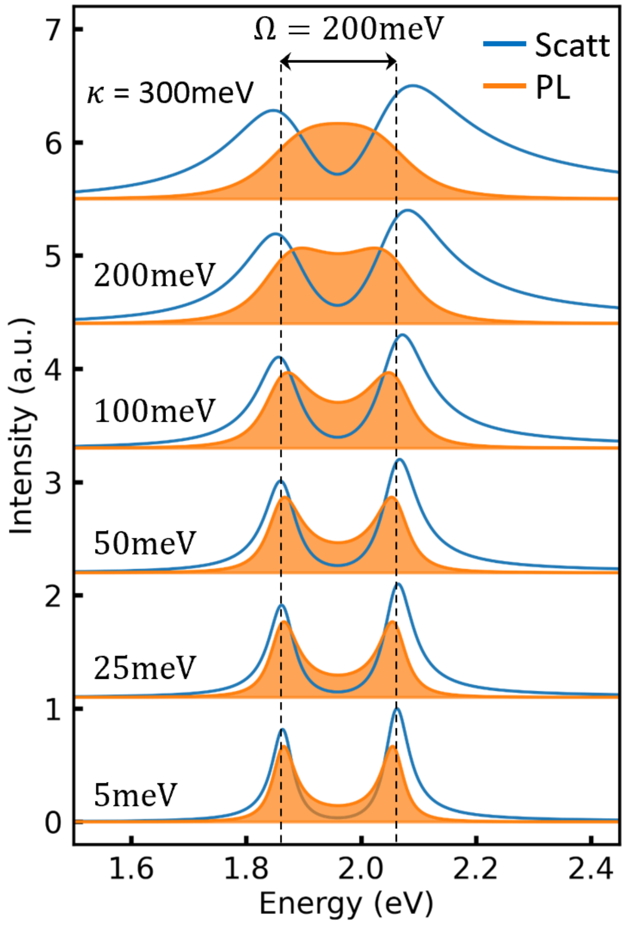

Fig. S11 | Simulated scattering and PL spectra for increasing cavity damping rate. Simulated scattering (blue) and PL spectra (orange) for increasing damping rate (κ) of the cavity (as labelled). The Rabi splitting energy and damping of the QD are set as Ω=200meV and γ=90meV.

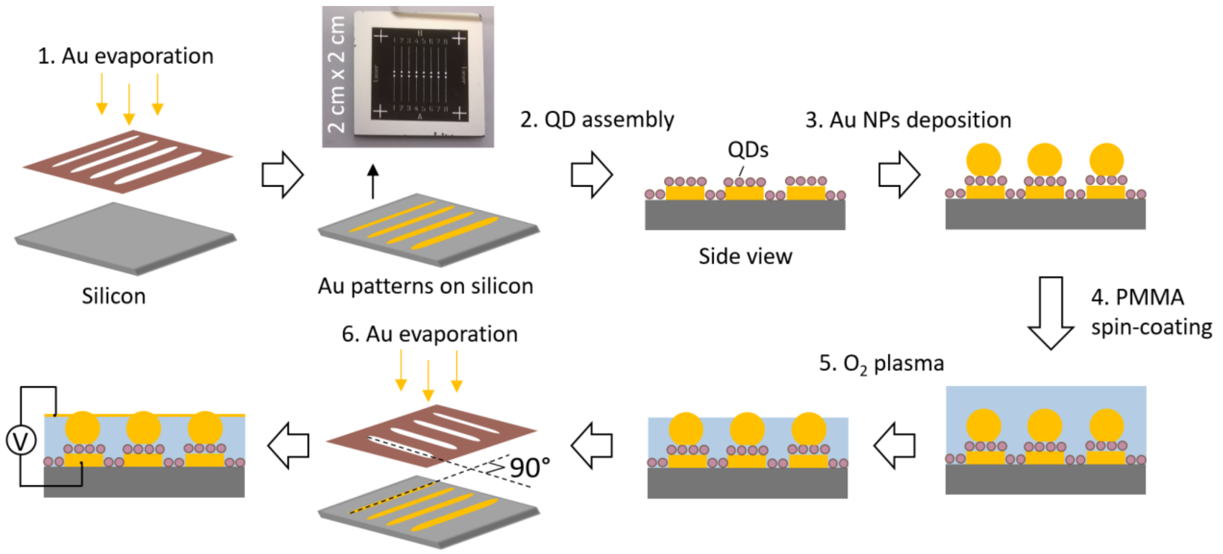

Fig. S12 | Schematic fabrication route for electroluminscent NPoM LED devices. Schematic fabrication route for electroluminscent NPoM LED devices.

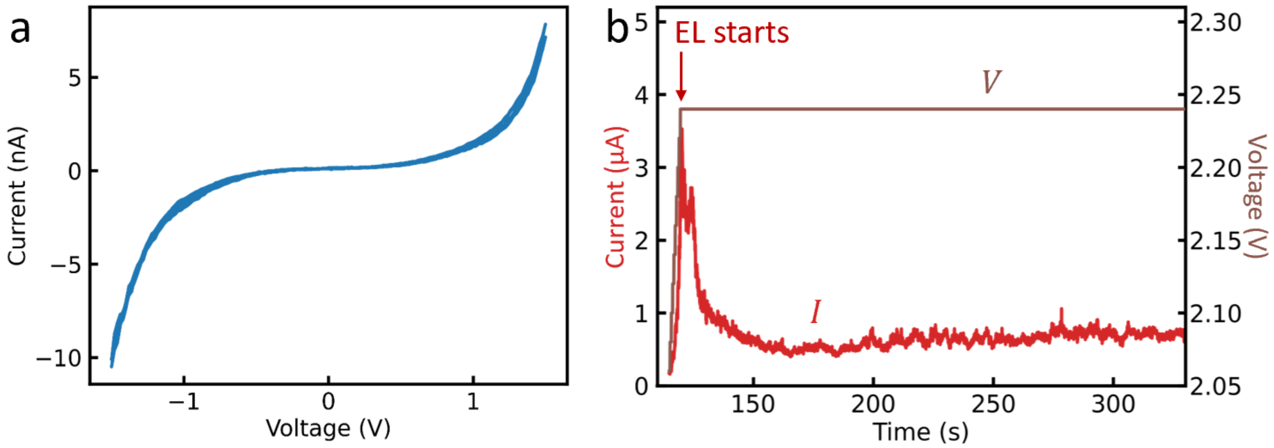

Fig. S13 | Electrical measurements of NPoM LED device. a, Repeated I-V measurements of an NPoM LED device below the voltage threshold for electroluminescence. **b,** Time dependent current fluctuations and applied voltage in the electroluminescent domain of an NPoM LED device. The current settles to 0.6 μA when the electroluminescence switches on at 2.24V.

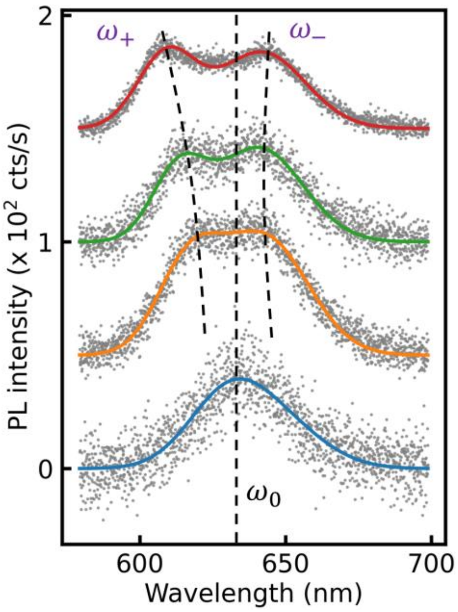

Fig. S14 | Time dependent electroluminescence fluctuations from an active NPoM LED device. Time dependent electroluminescence fluctuations from an active NPoM LED device. Time increases from bottom to top at 0, 13, 19, and 30s.

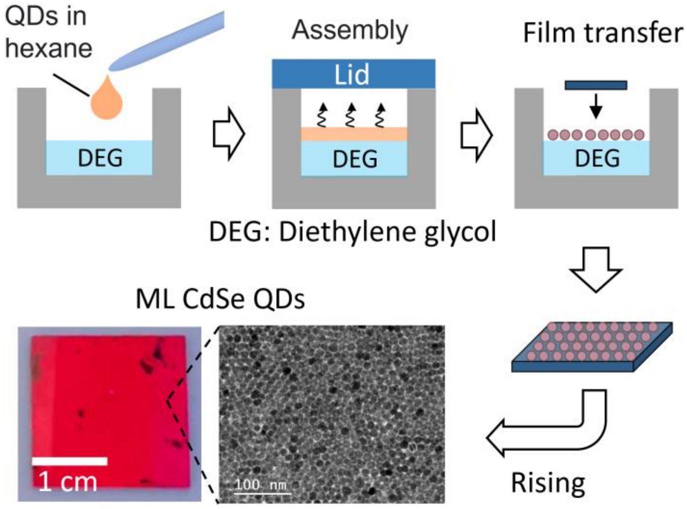

Fig. S15 | The schematic of monolayer QDs assmebly using liquid-air interface approach.

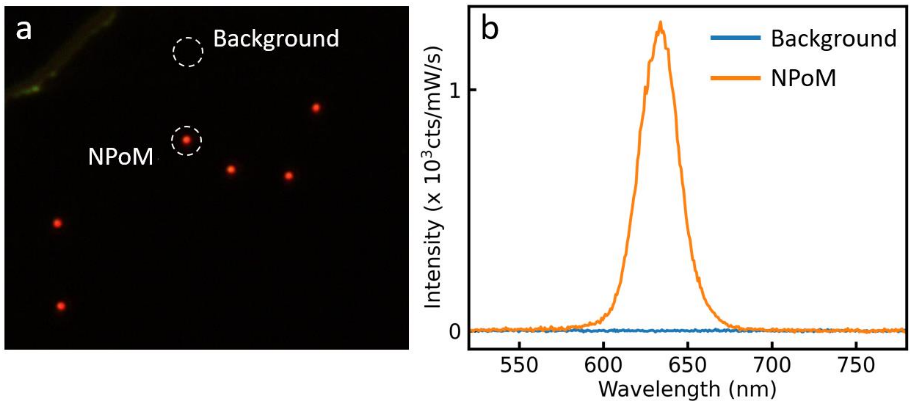

Fig. S16 | QD photoemission from monolayer QD film with and without NPoM cavity. a,b, Comparison of QD photoemission acquired from a monolayer QD film both with (orange) and without (blue) the presence of an NPoM nanocavity (see dashed circles in(a)), after one hour of O_2 plasma treatment.

References

1. Dement DB, Puri M, Ferry VE. Determining the complex refractive index of neat CdSe/CdS quantum dot films. *J. Phys. Chem. C* **122**, 21557-21568 (2018).
